# Avoiding Malnutrition in the Era of Glucagon-Like Peptide-1 Medications: Emerging Evidence and Opportunities for Integrated Nutrition Care

**DOI:** 10.1016/j.tjnut.2026.101684

**Published:** 2026-06-20

**Authors:** Paola Mogna-Peláez, Marta Guasch-Ferré

**Affiliations:** 1 Department of Public Health, Section of Epidemiology, Faculty of Health and Medical Sciences, University of Copenhagen, Copenhagen, Denmark; 2 Novo Nordisk Foundation Center for Basic Metabolic Research, University of Copenhagen, Copenhagen, Denmark; 3 Department of Nutrition, Harvard T.H. Chan School of Public Health, Boston, MA, United States

**Keywords:** nutritional assessment, dietary intake, antiobesity medications, glucagon-like peptide receptor agonists, sarcopenia

## Abstract

Glucagon-like peptide-1 (GLP-1)–based therapies have transformed obesity treatment, resulting in significant weight loss and broad metabolic improvements. However, their rapid adoption has outpaced the development of adequate nutritional support systems, leaving many patients without dietary guidance despite experiencing profound reductions in appetite and food intake. People often drastically cut their energy consumption, risking inadequate protein intake, micronutrient deficiencies, low fiber intake, and loss of lean mass, consequences that are largely preventable with proper guidance. Yet, in clinical practice, integrated nutritional assessment is rarely provided, despite obesity societies recognizing its importance. This perspective synthesizes emerging evidence on the nutritional risks associated with GLP-1–based therapy, examines the gap between guideline recommendations and real-world practice, and argues that integrated nutritional care must become a core (not optional) part of GLP-1–based obesity treatment. We emphasize the role of registered dietitians and behavioral health professionals in maintaining nutrient adequacy, reducing gastrointestinal side effects, and preserving lean mass. Additionally, we identify key research priorities: developing validated nutritional screening tools, defining evidence-based nutrient targets, characterizing high-risk patient profiles, and researching the long-term outcomes of integrated nutrition and pharmacotherapy. As GLP-1 prescriptions reach millions of patients across diverse and often under-resourced clinical settings, integrating nutrition into this care model is not just advisable; it is urgent.

## Introduction

The arrival of glucagon-like peptide-1 (GLP-1)–based medications, either alone or in combination with glucose-dependent insulinotropic polypeptide agonists, marks a significant breakthrough in obesity treatment [1]. Despite decades of increasing obesity rates and ongoing efforts to curb them, effective weight-loss strategies have remained difficult for many. These medications have revolutionized the field by providing substantial weight loss and numerous metabolic benefits, such as improved glycemic control, better management of type 2 diabetes (T2D), increased insulin sensitivity, lower blood pressure, improved lipid profiles, and meaningful improvement in metabolic dysfunction-associated steatotic liver disease (MASLD) spectrum, including reduction in hepatic steatosis and, in patients with established steatohepatitis, histologic improvement and fibrosis regression in metabolic dysfunction-associated steatohepatitis [[2], [3], [4]]. The emergence of GLP-1 therapies has changed both clinical practice and patient expectations [1,5].

However, this significant medical advance also introduces new and underrated responsibilities. Because these medications heavily suppress appetite and slow gastric emptying, many people, particularly those managing treatment without proper guidance, substantially reduce their food intake [[6], [7], [8]]. Although this can lead to rapid weight loss, it also risks unintended nutritional consequences, including inadequate protein and micronutrient intake, low dietary fiber intake, and overall insufficient energy intake [9,10]. These risks are especially prevalent in real-world settings where integrated nutritional oversight is often absent. In many clinical contexts, prescriptions are given without a comprehensive dietary assessment or the time and resources needed to provide tailored nutritional support [6,11,12].

We argue that the rapid and widespread adoption of GLP-1, now reaching millions of patients across primary care, telemedicine, and commercial weight-loss settings, has created a predictable nutritional risk that requires a systematic, evidence-based response. We describe this as integrated nutrition care throughout this perspective: a comprehensive, individualized approach that includes baseline dietary assessment, evidence-based nutrient targets, ongoing monitoring of nutritional status and body composition, and registered dietitian-led counseling. This stands in contrast to the brief, generic dietary advice that characterizes most GLP-1 prescribing encounters.

For many patients, nutrition is not being adequately addressed during GLP-1 treatment, and this gap is preventable. Importantly, practice patterns in this area are still being formed: the decisions made now about how to integrate (or not integrate) nutritional care into GLP-1 prescribing will shape standards for years to come. This perspective first examines the unintended nutritional consequences of appetite suppression, then considers the gaps between clinical guidelines and everyday care, and finally proposes a framework and research priorities for establishing nutrition as a central pillar of GLP-1-based treatments, not an optional adjunct, but an evidence-based necessity.

### The unintended nutritional consequences of appetite suppression

GLP-1–based therapies partly work by reducing appetite and slowing gastric emptying. As a result, many people naturally eat significantly less. A randomized trial of oral semaglutide found total daily energy intake reduced by ∼39% compared with placebo [13], consistent with broader data across multiple GLP-1 agents showing reductions of 16% to 39% [11]. Although this decrease in energy intake supports weight loss, it also presents significant nutritional challenges when it happens without proper guidance [9,13,14]. Many patients struggle to meet basic dietary needs, especially when appetite is very low or when nausea makes eating difficult [9,14].

Without structured support, protein intake often falls critically short of weight-loss targets: in a cross-sectional study of adults using GLP-1 receptor agonists (RAs), only 43% met the minimum protein threshold of 1.2 g/kg/d, although the majority failed to meet dietary reference intakes for vitamin D, potassium, magnesium, and iron[14]. In a large retrospective analysis of >460,000 GLP-1 RA users, nutritional deficiencies were documented in 12.7% within 6 mo and 22.4% within 12 mo of treatment initiation [9]. Clinical manifestations may include fatigue, hair thinning, and persistent gastrointestinal (GI) symptoms, findings documented across observational and clinical studies [6,15]. Such observations underscore the importance of systematically monitoring dietary adequacy to prevent potential short- and long-term nutritional consequences [8,12].

Low protein intake significantly increases the risk of disproportionate lean mass loss during weight loss. Evidence from clinical trials and network meta-analyses shows that lean mass makes up ∼25% of total weight lost with GLP-1–based therapy, a proportion that may be partly adaptive and is not specific to this drug class, but the actual amount of lean mass lost can be substantial depending on the level of weight reduction achieved [16]. Importantly, disproportionate loss of fat-free mass can harm metabolic health and physical function independently of total weight loss, and this risk is especially high in older adults, where even small decreases in muscle mass may lead to functionally significant sarcopenia [[17], [18], [19]]. Long-term data further indicate that muscle strength may decline gradually beyond the first year of treatment, even if mass measurements seem stable, highlighting the need for continuous monitoring [17]. If undernutrition persists, these changes can progress to overt malnutrition [18,19]. Low fiber and fluid intake may further exacerbate GI problems such as constipation, bloating, and nausea, creating a cycle in which patients eat even less [7,14,20].

Beyond the clinical setting, these risks may be further amplified by emerging changes in the food environment. Commercial responses to the growing use of GLP-1 therapies include reduced portion sizes in restaurant settings and the marketing of “GLP-1–friendly” meals tailored to individuals with suppressed appetite [21,22]. Although such approaches may align with reduced energy intake, their nutritional adequacy is likely to be largely unregulated and rarely evidence-based. Consuming smaller portions of nutritionally incomplete meals may inadvertently exacerbate deficiencies in protein and micronutrients. In this context, the primary challenge is not portion size but nutrient density, a distinction that is often overlooked in commercial messaging. Without appropriate guidance from qualified nutrition professionals, these market-driven adaptations may create a false sense of dietary adequacy while failing to address underlying nutritional risk.

Although these consequences can affect any patient, nutritional risk during GLP-1 therapy is not always uniform. It is significantly increased by factors such as age, baseline nutritional status, the reason for treatment, and the burden of comorbidities. Older adults and frail individuals are especially vulnerable: decreased physiological reserve and less efficient muscle protein synthesis mean they are less capable of compensating for inadequate intake, putting them at higher risk of both malnutrition and sarcopenia [19,23]. Patients on GLP-1 therapy for T2D may already face increased micronutrient deficiencies (notably vitamin B12, particularly in those taking metformin, and vitamin D), whereas those with MASLD may experience impaired absorption of fat-soluble vitamins and disrupted liver function affecting nutrient metabolism [9]. Moreover, patients starting GLP-1 therapy in preparation for surgery are another high-risk group, as pre-existing nutritional issues may worsen due to appetite suppression during the perioperative period [24]. Recognizing these different risk profiles is crucial for targeting nutritional screening where it is most urgently needed.

### Gaps between guidelines and real-world practice

In real-world practice, many patients begin GLP-1 therapy with little or no nutritional support. A large proportion of prescriptions come from primary care, telemedicine services, and commercial weight-loss clinics, where time and resources for detailed nutrition counseling are limited [8,12]. The rapid expansion of telemedicine and the increasing availability of compounded formulations may further limit medical oversight, increasing the likelihood that GLP-1 therapies are initiated without assessment of baseline dietary intake, nutritional status, or potential risks [7,12,20].

Essential aspects such as baseline dietary habits, micronutrient concentrations, protein consumption, eating behaviors, and potential barriers to proper nutrition are rarely examined [25]. This was markedly illustrated by a recent retrospective study that found patients taking GLP-1 agonists before elective joint surgery had ∼7 times the odds of severe malnutrition compared to non-users, despite the absence of routine nutritional screening [24]. This approach does not align with recommendations from obesity medicine societies, which stress individualized nutrition counseling as a core component of pharmacologic treatment [12]. Without this support, patients are left to interpret vague advice on their own, often during a period when their appetite is significantly reduced, and eating becomes more challenging.

In practice, this means individuals must guess what and how much to eat, or assume that pre-existing dietary habits can continue, often leading to inadequate intake and preventable nutritional issues [6,24]. This disconnection between clinical guidelines and everyday care underscores a critical opportunity to better align nutritional recommendations with routine GLP-1 prescribing [12].

### The role of nutrition as a central component of GLP-1–based care

A comprehensive nutritional assessment should therefore be part of every patient’s care plan when starting GLP-1 therapy. Key components include establishing a baseline nutritional profile, reviewing current eating patterns, and identifying potential risks before treatment begins, a step supported by evidence that GLP-1 RA users consistently show caloric reductions of 16% to 39% and widespread micronutrient inadequacies from early in treatment [11].

Throughout therapy, clinicians should monitor protein intake. Provisional targets (largely extrapolated from the broader weight-loss and sarcopenia literature rather than GLP-1–specific randomized trials) suggest ∼1.2 to 1.5 g/kg/d for younger, otherwise healthy individuals, and 1.6–2.0 g/kg/d for older adults, those at risk of sarcopenia, or those experiencing rapid rates of weight loss [12,18,19]. Importantly, these ranges require validation in GLP-1–specific studies before they can be considered established recommendations for this population. Ensuring sufficient micronutrient intake is equally vital, as deficiencies can develop quickly when appetite is low, or food variety decreases [8]. Additional factors, such as fiber intake, hydration, and tolerance to different food textures, play an important role in minimizing GI side effects [7,14,20].

Early treatment phases, especially the first 3 mo, may require a structured progression of food types and portion sizes to match the patient’s changing appetite and digestive comfort [8,9,12]. In this context, it is also crucial to recognize that nutritional support should be adaptable rather than rigid, adjusting to the patient’s treatment stage, medication dosage, and baseline risk factors. During the initial phase, appetite suppression often occurs while GI symptoms are most severe; the primary nutritional goal at this point is to foster sustainable eating habits, manage symptom-related food aversions, and prevent abrupt caloric restriction that could lead to significant lean mass loss [16]. As dosages increase and appetite reduction worsens, nutritional monitoring should become more rigorous, with greater focus on protein deficiency and early signs of micronutrient shortages. At maintenance doses, when the most weight loss occurs, the risk of ongoing lean mass loss and micronutrient deficiencies rises; regular reassessment of body composition becomes particularly important at this stage [16,17,26]. Higher doses are independently associated with greater lean mass loss, underscoring the importance of tailoring nutritional care to the pharmacologic intensity rather than maintaining a constant regimen throughout treatment [16,17]. Throughout all phases, patients with the highest initial risk (such as older adults, those with pre-existing low lean mass or sarcopenia, and individuals experiencing rapid weight loss) require the most proactive and intensive nutritional support [8,12].

Translating nutritional recommendations into practice, however, requires explicitly addressing the challenge of patient adherence. GLP-1–induced nausea, early satiety, and food aversions (which are typically most pronounced during dose initiation) can make it genuinely difficult for patients to follow dietary guidance, even when it is clearly provided [19]. Behavioral strategies can meaningfully improve adherence in this context: consuming small, frequent, nutrient-dense meals; prioritizing protein-rich foods at the beginning of eating occasions when satiety is least pronounced; adapting food temperature and texture to improve GI tolerance; and timing meals away from periods of peak nausea are practical approaches supported by dietary management literature [19,27]. Psychological and behavioral health support, including motivational interviewing, cognitive-behavioral approaches to food-related avoidance, and structured goal setting, provides an additional layer of care that is particularly relevant for patients with pre-existing disordered eating behaviors or high emotional reactivity to appetite changes [28,29]. Integrating behavioral health professionals into the GLP-1 care team is therefore not peripheral but necessary for sustainable nutritional adherence.

**FIGURE 1:**
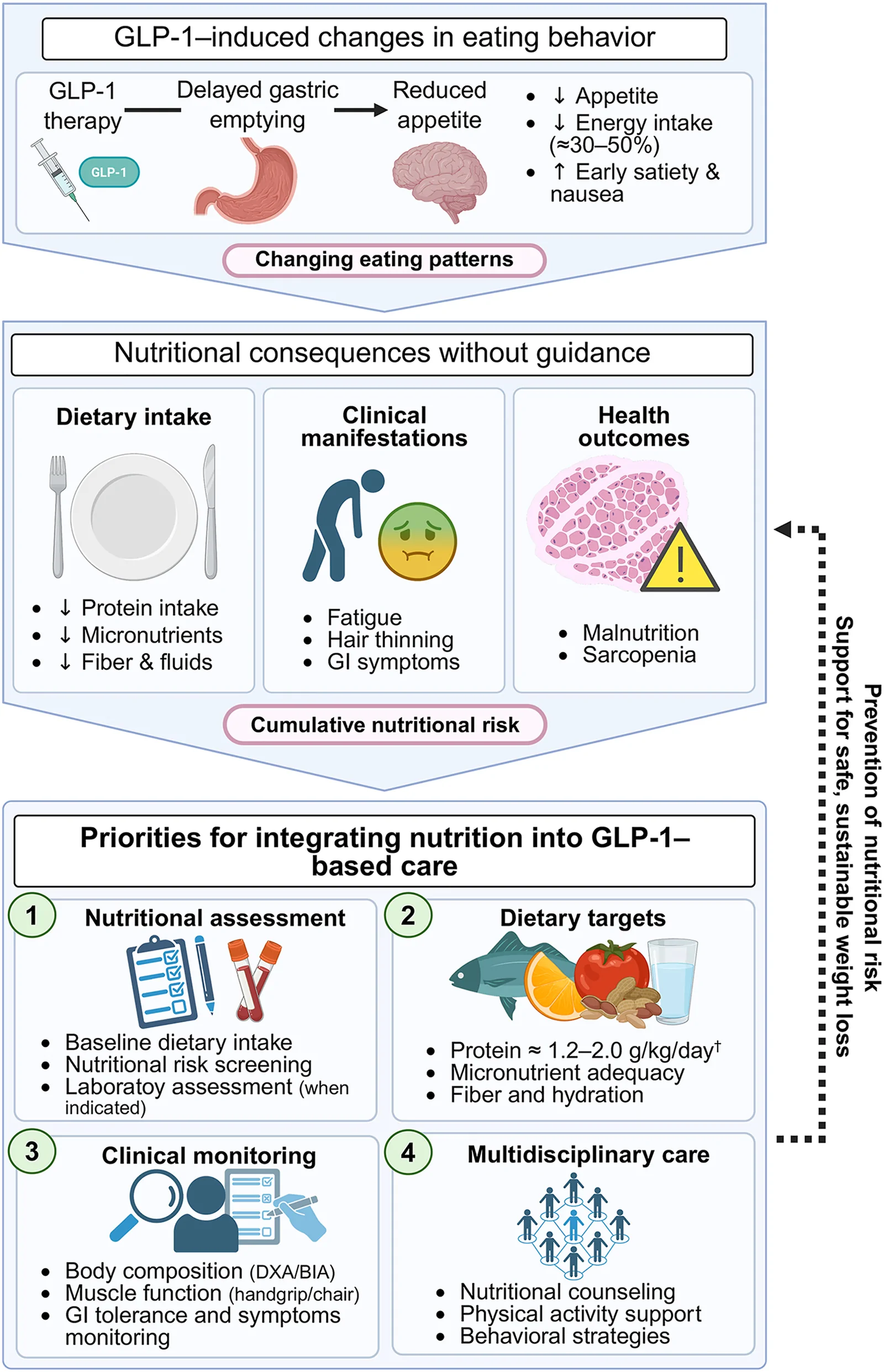
Conceptual framework for integrating nutrition into GLP-1–based obesity care. GLP-1–based therapies reduce appetite and slow gastric emptying, leading to marked reductions in energy intake (≈30–50%) and changes in eating behavior. In the absence of integrated nutritional guidance, these changes may result in inadequate intake of protein, micronutrients, fiber, and fluids, contributing to cumulative nutritional risk and adverse clinical outcomes, including malnutrition and sarcopenia. The lower panel outlines key priorities for integrating nutrition into GLP-1–based care: *1*) baseline nutritional assessment and risk screening; *2*) individualized dietary targets to support protein adequacy and overall nutrient intake; *3*) ongoing clinical monitoring of body composition, using dual-energy X-ray absorptiometry (DXA) or bioelectrical impedance analysis (BIA), and muscle function (e.g., handgrip strength or chair-rise testing), alongside gastrointestinal (GI) tolerance and nutritional status; and *4*) multidisciplinary care incorporating nutrition, physical activity, and behavioral support. Protein targets are provisional and patient-dependent, extrapolated from weight-loss and sarcopenia literature; higher intakes (1.6–2.0 g/kg/d) may be most relevant for older adults and individuals at risk of sarcopenia. GLP-1–specific evidence remains limited and requires further validation. This conceptual framework synthesizes evidence from prior studies on GLP-1–induced changes in appetite and dietary intake, nutritional risk, and muscle health [9,[12], [13], [14],^17^,^18^,^24^,^26^,^28^,^30^]. GLP-1, glucagon-like peptide-1.

When possible, body composition assessment using dual-energy X-ray absorptiometry (DXA) or, as a more accessible clinical option, bioelectrical impedance analysis (BIA), along with muscle function tests like handgrip strength or chair-rise testing, should be included in this monitoring. These methods provide objective, long-term data on lean mass changes that weight-based outcomes alone cannot reflect [17,26]. Regular screening for signs of nutritional deficiencies, such as hair loss, fatigue, excessive dietary restriction, or abnormal laboratory findings, may facilitate earlier identification of nutritional compromise and timely clinical intervention [6,8,9]. These key elements are integrated into a conceptual framework outlining nutritional priorities for patients receiving GLP-1–based therapies (Figure 1 [9,[12], [13], [14],^17^,^18^,^24^,^26^,^28^,^30^]).

### Research gaps and future directions

The growing use of GLP-1 therapies highlights several important gaps in the current nutritional evidence base. Most available evidence on nutrition-related outcomes in GLP-1 users derives from retrospective analyses, cross-sectional studies, and narrative reviews; randomized controlled trials with nutrition as a primary or co-primary outcome remain scarce [9,11,14]. This limitation weakens current recommendations and underscores the urgency of purpose-built trials in which dietary intake, body composition, micronutrient status, and functional outcomes are treated as endpoints of equal priority to weight loss and glycemic control.

A priority is the development and validation of practical tools to assess nutritional risk in patients using these medications [29,31]. This includes simple screening questionnaires, guidance for clinicians on evaluating dietary intake, and improved education for prescribers about the nutritional challenges that may arise during treatment. Integrating other health professionals, such as registered dietitians, nutritionists, physical activity specialists, and psychologists, into routine care is also essential to provide patients with comprehensive support [30,31].

Future research should aim to define optimal nutritional targets for individuals using GLP-1 therapy. This includes GLP-1–specific randomized trials to define optimal protein intake by patient profile (age, rate of weight loss, and baseline sarcopenia risk), guidance on when supplementation may be necessary, and strategies for monitoring micronutrient status over time [8,12].

Critically, future studies should routinely incorporate body composition assessments (DXA or BIA) and standardized muscle function measures (including handgrip strength or chair-rise testing) as coprimary or key secondary endpoints alongside weight loss and glycemic outcomes. Current evidence is hindered by heterogeneity in measurement approaches and the near-universal absence of functional endpoints, making it difficult to draw firm conclusions about the clinical significance of lean mass changes or to compare findings across trials [16,26]. Identifying which patients are most vulnerable to malnutrition, muscle loss, or deficiencies will help clinicians intervene earlier and tailor care more effectively.

In addition, long-term studies examining the combined impact of integrated nutrition support and GLP-1 pharmacotherapy remain limited. Understanding how these elements interact and how they influence weight maintenance, metabolic health, and quality of life will be crucial for improving future treatment models. Mechanistic research is needed to determine whether adequate nutritional support potentiates or attenuates GLP-1–mediated effects on body composition and metabolic outcomes, a question with direct implications for optimizing combined treatment strategies.

Furthermore, research on implementation is vital to understand how integrated nutrition care can be effectively delivered across different clinical settings, from specialist obesity clinics with multidisciplinary teams to primary care and telemedicine environments where access to registered dietitians is limited. It is also essential to investigate whether simplified, scalable models can achieve comparable nutritional outcomes. Finally, there is an urgent need for implementation studies on strategies to support dietary adherence during GLP-1–induced GI symptoms, including comparing the effectiveness of behavioral coaching, digital health platforms, and symptom-guided meal planning in improving nutritional results.

In conclusion, GLP-1–based therapies represent a true breakthrough in obesity treatment, but their full clinical impact depends on systematically addressing the nutritional vulnerabilities they create. This perspective emphasizes that integrated nutritional care must become a core, non-negotiable part of GLP-1 prescribing, not something optional or dependent on available resources, but a structured clinical process from the start of treatment. The evidence reviewed here identifies who is most at risk (older adults, patients with T2D, MASLD, or preoperative needs), when risk is highest (during dose titration and at maintenance doses), and what requires specific monitoring (protein intake, micronutrient concentrations, body composition via DXA or BIA, and muscle function). Behavioral health support is equally vital to help patients stay adherent when appetite suppression and GI symptoms make eating difficult. Importantly, prescribing norms for GLP-1 therapies are still evolving. Decisions made now about whether nutritional assessment, dietitian involvement, and body composition monitoring become standard or remain exceptions shape the future of clinical care. Building the evidence through purpose-specific trials that include nutritional primary outcomes while simultaneously improving current practices is essential. The cost of inaction is a future in which patients lose lean mass, develop micronutrient deficiencies, and face outcomes that could have been prevented with evidence-based nutritional care.

## Author contributions

The authors’ responsibilities were as follows – PM-P: conceived the perspective and drafted the initial manuscript; MG-F: provided intellectual input, contributed to subsequent revisions, and oversaw the conceptual development of the piece. Both authors read and approved the final manuscript.

## Declaration of AI and AI-Assisted Technologies in the Writing Process

During the preparation of this work, the author(s) used Claude to assist with manuscript review after the manuscript had already been drafted, Grammarly Premium to assist with English-language proofreading, and BioRender.com to create Figure 1. After using these tools/services, the author(s) reviewed and edited the content as needed and take(s) full responsibility for the content of the publication.

## Funding

PM-P and MG-F are supported by the Novo Nordisk Foundation Center for Basic Metabolic Research, which is an independent research center at the University of Copenhagen, partially funded by an unrestricted donation from the Novo Nordisk Foundation (NNF23SA0084103). The views expressed in this manuscript are solely those of the authors and do not necessarily represent the positions of their affiliated institutions.

## Conflict of interest

The authors report no conflicts of interest.
